# Associative Patterns Between Iron Deficiency Anemia and Febrile Seizures in the Five to 60 Months Age Group: A Comprehensive Systematic Review

**DOI:** 10.7759/cureus.56470

**Published:** 2024-03-19

**Authors:** Saloni Bakkannavar, Youmna Faheem, Amisha Jaiswal, Kainaat Shergill, Kusalik Boppana, Naiela E Almansouri, Pousette Hamid

**Affiliations:** 1 Pediatrics, California Institute of Behavioral Neurosciences & Psychology, Fairfield, USA; 2 Medicine, Jagadguru Jayadeva Murugarajendra Medical College, Davanagere, IND; 3 Medicine, Ras Al Khaimah Medical & Health Sciences University, Ras Al Khaimah, ARE; 4 Medicine, New Medical Center Royal Hospital, Abu Dhabi, ARE; 5 Internal Medicine, California Institute of Behavioral Neurosciences & Psychology, Fairfield, USA; 6 Surgery, California Institute of Behavioral Neurosciences & Psychology, Fairfield, USA; 7 Medicine, Maharishi Markandeshwar Institute of Medical Sciences & Research, Ambala, IND; 8 Gastroenterology, California Institute of Behavioral Neurosciences & Psychology, Fairfield, USA; 9 Internal Medicine, University of Tripoli, Tripoli, LBY; 10 Neurology, California Institute of Behavioral Neurosciences & Psychology, Fairfield, USA

**Keywords:** simple febrile seizure, children, febrile convulsion, febrile seizures, iron deficiency anemia (ida)

## Abstract

Febrile seizures (FS) are commonly seen in younger age groups. The cause of seizures is multifactorial, including viral illnesses, certain vaccines such as MMR (measles, mumps, rubella), family history of FS, and certain mineral deficiencies like zinc. Iron deficiency anemia (IDA) is the most common cause of anemia in children of the same age group. The systematic review was conducted according to the Preferred Reporting Items for Systematic Reviews and Meta-Analysis (PRISMA) guidelines. This review aimed to investigate the correlation between IDA and fever convulsions. A systematic literature search was conducted using PubMed and Google Scholar databases for studies published between January 2013 and September 2023. The following keywords were used to search the articles: “children”, “febrile seizures”, and “iron deficiency anemia”, using all possible combinations and using the word “and” between them. Following the inclusion and exclusion criteria application, we included 23 case-control studies written in the English language in this study. Quality assessment of studies was done using the Newcastle Ottawa Scale.

## Introduction and background

Convulsions developed due to temperatures above 38°C (100.4°F) are termed as febrile seizures (FS). These seizures are not caused by any central nervous system infections (CNS) or any metabolic abnormalities. Essentially, it occurs in individuals without a prior history of afebrile seizures [1]. It is the most commonly observed seizure type in children under five years of age [1]. Roughly one-third of children who have one FS may experience a second. Among children with simple FS, 1% to 2% may develop epilepsy. In contrast, those with complex FS have a higher risk, with 6% to 8% later being diagnosed with epilepsy [2].

Iron deficiency and iron deficiency anemia (IDA) are pervasive on a global scale. Roughly 30% to 40% of people worldwide suffer from IDA, with the majority residing in developing nations [3]. IDA has stood as the primary contributor to the healthcare burden in India over the last 10 years [4]. Iron deficiency can adversely affect the developing brain in multiple ways. It can impair hippocampal neuron growth and disrupt the myelination process, which insulates nerve fibers for efficient signaling. This deficiency can also impact the metabolism of neurotransmitters such as monoamines and aldehyde oxidase, resulting in lower neurotransmitter levels and a potential decrease in the seizure threshold. Moreover, low serum ferritin levels in conjunction with fever can compound the adverse effects on the brain, potentially leading to seizures [5]. Notably, it's worth mentioning that the peak ages for FS and IDA coincide [5]. While certain studies identified a link between FS and anemia resulting from iron deficiency [6-16], other research yielded divergent findings [17-20]. The primary objective of this study is to find the association between IDA and FS in children by doing a systematic review of the literature of the last 10 years. The secondary objective is to understand the significance of the iron profile in FS.

## Review

Search strategy 

This study is a systematic review done in accordance with Preferred Reporting Items for Systematic Review and Meta-analyses (PRISMA) guidelines.

Data Sources and Search Strategy

We performed a literature review using PubMed and Google Scholar databases. It included all research published in the last 10 years (2013-2023), up to and including 18 September 2023. Using a combination of Medical Subject Heading (MeSH) and keywords such as “iron deficiency anemia”, "febrile seizures", and "simple febrile seizures”, we employed a Boolean approach to search for relevant publications on the pattern of IDA and FS.

Details of the search strategy are listed in Table 1.

**Table 1:** Search strategy

Database	Search strategy
PubMed	("Child"[MeSH Terms] OR "Child" OR "Children") AND ("Anemia, Iron-Deficiency"[MeSH Terms] OR "Iron-Deficiency Anemia" AND ("Seizures, Febrile” OR "Convulsions, Febrile" OR "Febrile Convulsion Seizure" OR "Febrile Fit" OR "Febrile Seizures" OR "Fever Convulsion" OR "Fever Seizure" OR "Pyrexial Convulsion" OR "Pyrexial Seizure" OR "Seizure, Febrile, Complex" OR "Seizure, Febrile, Simple" OR "Seizures, Febrile")
Google Scholar	"Iron deficiency anemia" and "febrile seizure"

Study Selection and Eligibility Criteria

Articles extracted from each database were compiled and duplicates were removed using Mendeley Reference Manager (Elsevier, Amsterdam, Netherlands) and manually. We conducted title and abstract screening to discern articles of relevance. Two independent authors independently examined the selected studies' full-text articles. Any disparities in data interpretation were resolved through deliberation and mutual agreement among the authors.

Inclusion Criteria

The inclusion criteria were as follows: (1) Observational studies; (2) Case definition - febrile seizures; (3) Control definition - febrile illness without seizures; (4) Sample size >100; (5) Free full-text articles in English.

Exclusion Criteria

Exclusion criteria were set as follows: (1) Randomized controlled trial, meta-analysis, systematic review, case reports, case series; (2) Animal studies; (3) Sample size <100; (4) Control definition - healthy controls (no febrile illness); (5) Febrile illness due to CNS infections or metabolic disease; (6) Seizures due to electrolyte imbalance and neurological disease; (7) Studies including children taking iron supplements.

Data extraction

The following dataset was compiled, encompassing author names, publication years, geographical study locations, sample sizes, types of FS under consideration, measured parameters, ultimate research outcomes, and concluding statements.

Quality assessment

The Newcastle Ottawa Scale was employed for evaluating the quality of the chosen articles and articles meeting more than or equal to six out of nine stars were included. Table 2 demonstrates the quality assessment of the studies included.

**Table 2:** Quality assessment of the studies

Author	Is the case definition adequate?	Representativeness of the cases	Selection of controls	Definition of controls	Comparability of cases and controls based on the design or analysis	Ascertainment of exposure	Same method of ascertainment for cases and controls	Non-response rate	Quality score
Basavaraj et al., 2021 [5]	1	1	1	1	1	1	1	0	7
Sharma et al., 2018 [6]	1	1	1	1	1	1	1	0	7
Madavi et al., 2021 [7]	1	1	1	1	1	1	1	0	7
Ghasemi et al., 2014 [8]	1	1	1	1	2	1	1	0	8
Bhat et al., 2020 [9]	1	1	1	1	1	1	1	0	7
Sharif et al., 2015 [10]	1	1	1	1	1	1	1	0	7
Gaballah et al., 2022 [11]	1	1	1	1	1	1	1	0	7
Agarwal et al., 2016 [12]	1	1	1	1	1	1	1	0	7
Sit et al., 2016 [13]	1	1	1	1	1	1	1	0	7
Saleem et al., 2020 [14]	1	1	1	1	1	1	1	0	7
Malla et al., 2015 [15]	1	1	1	1	1	1	1	0	7
Subbarao et al., 2019 [16]	1	1	1	1	1	1	1	0	7
Tripathy et al., 2020 [17]	1	1	1	1	1	1	1	0	7
Awais et al., 2022 [18]	1	1	1	1	0	1	1	0	6
Jang et al., 2019 [19]	1	1	1	1	1	1	1	0	7
Yarigarravesh et al., 2021 [20]	1	1	1	1	1	1	1	0	7
Chaudhary et al., 2021 [21]	1	1	1	1	1	1	1	0	7
Shaikh et al., 2018 [22]	1	1	1	1	1	1	1	0	7
Mallela et al., 2022 [23]	1	1	1	1	2	1	1	0	8
Kumari et al., 2023 [24]	1	1	1	1	1	1	1	0	7
Sarmad et al., 2020 [25]	1	1	1	1	1	1	1	0	7
Shankar et al., 2019 [26]	1	1	1	1	1	1	1	0	7
Elkafafy et al., 2021 [27]	1	1	1	1	1	1	1	0	7

Results

The initial search approach resulted in 524 and nine records once filters were used in Google Scholar and PubMed, respectively. After employing Mendeley software and manual review, 33 duplicates were eliminated, resulting in a pool of 491 articles for the initial screening. Subsequently, after evaluating titles and abstracts, 125 articles were identified for retrieval. A total of 81 full-text literature was assessed for eligibility. Of them, 58 articles were excluded for the following reasons: systematic review, literature review, no data on anemia, no data on p-value, no control, poor quality, improper result, and irrelevant data.

**Figure 1:**
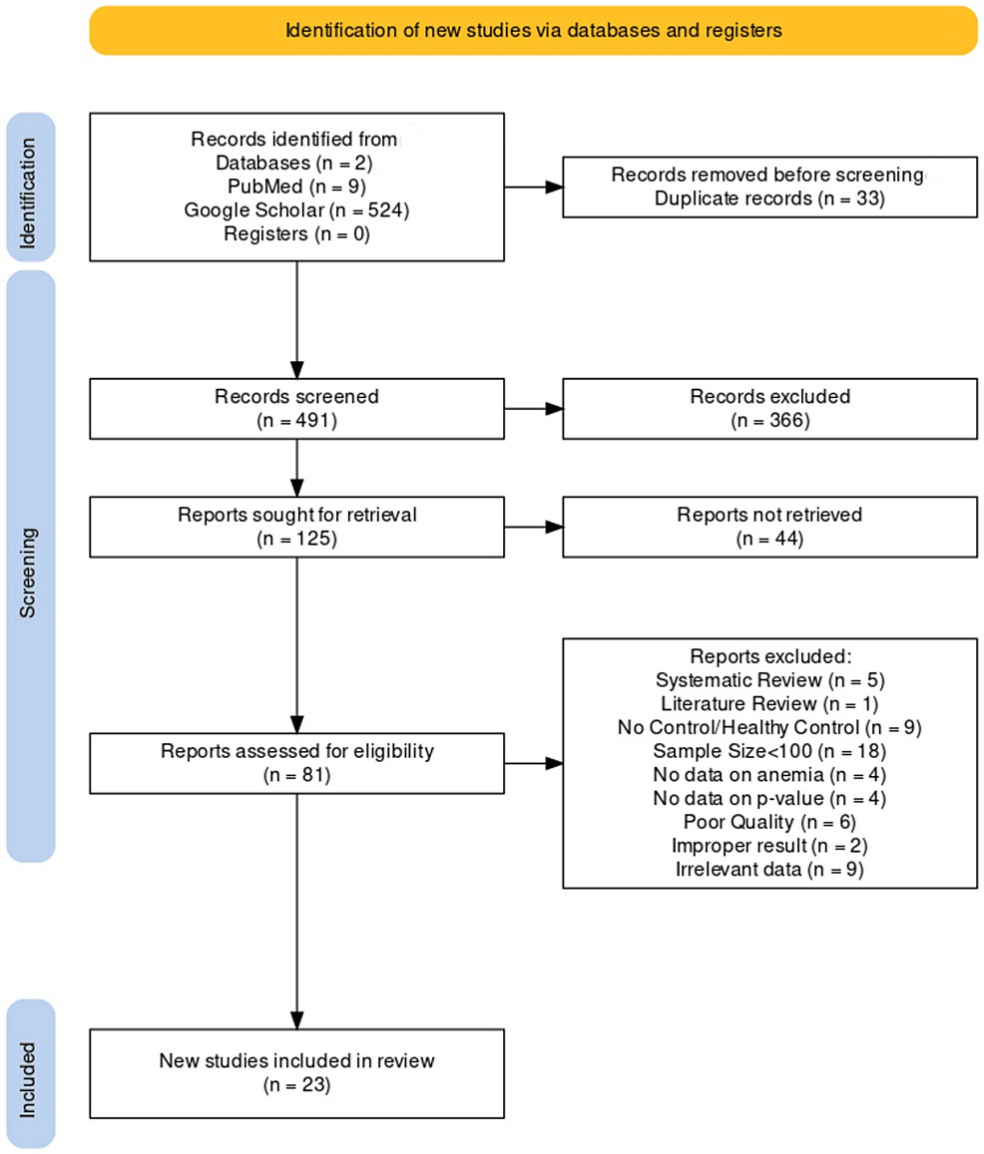
PRISMA flowchart for data extraction process PRISMA: Preferred Reporting Items for Systematic Review and Meta-analyses

Figure 1 demonstrates the flow chart of the process of inclusion of articles.

Table 3 highlights the characteristics of the studies including year, author, country in which studies were done, age group of children, sample size, types of FS, parameters measured, and conclusion of studies.

**Table 3:** Summary of all the studies in this systematic review Hb: Hemoglobin; MCV: Mean Corpuscular Volume; MCH: Mean Corpuscular Hemoglobin; MCHC: Mean Corpuscular Hemoglobin Concentration; RDW: Red Cell Distribution Width; Hct: Hematocrit; TIBC: Total Iron Binding Capacity; IDA: Iron Deficiency Anemia

Author and year	Country	Age	Sample size (case/ control)	Type of febrile seizures (FS)	Parameters measured and p-value between cases and controls	Conclusion
Basavaraj et al., 2021 [5]	India	6-60 months	50/50	Simple	Hb, MCV, MCH, MCHC, RDW, Transferrin saturation%, Serum Iron, Serum Ferritin <0.001; Transferrin-0.29	Significant association
Sharma et al., 2018 [6]	India	6-60 months	50/50	Both simple and complex	Hb-0.003; MCV-0.004; MCHC- 0.726; Hct, RDW <0.001; Serum Ferritin <0.001	Significant association
Madavi et al., 2021 [7]	Sri Lanka	6-60 months	60/60	Simple	Hb, MCH, MCHC <0.0001; MCV-0.006; Serum Iron-0.04; TIBC- 0.006; Serum Iron/TIBC-0.004	Significant association
Ghasemi et al., 2014 [8]	Iran	5-60 months	100/100	Both simple and complex	Hb-0.01, Hct-0.00, RBC-0.00, MCV-0.19, MCH-0.5, MCHC-0.88, Serum Ferritin-0.002, Serum Iron-0.05, TIBC-0.00, Transferrin saturation%-0.00, IDA-0.04	Significant association
Bhat et al., 2020 [9]	India	7-59 months	160/160	Simple	Hb-0.005; MCV <0.001; MCH-0.008; MCHC-0.04; RDW-0.02; TIBC-0.003; Transferrin <0.001; Serum Ferritin-0.008; IDA <0.0001	Significant association
Sharif et al., 2015 [10]	Iran	6-60 months	100/100	Not mentioned	Hb-0.06, Serum Iron-0.02, TIBC-0.0001, Transferrin Saturation-0.22, IDA-0.0005	Significant association
Gaballah et al., 2022 [11]	Egypt	6months-6 years	55/55	Simple	Hb-0.002; MCV-0.009; MCH-0.05; RDW-0.004; Serum Ferritin, TIBC, Anemia <0.001	Significant association
Agarwal et al., 2016 [12]	India	1-5 years	60/60	Simple	Mean Hb Level and MCV, RDW-0.02; MCH levels-0.07; Serum Ferritin and Serum Iron levels-0.01; TIBC value-0.01.	Significant association
Sit et al., 2016 [13]	India	6-60 months	50/50	Simple	Hb, MCV, MCH, Serum Ferritin <0.01, IDA <0.05	Significant association
Saleem et al., 2020 [14]	India	1-5 years	50/50	Simple	Hb <0.01; MCV <0.04; MCH-0.06; RDW <0.04; Serum Ferritin, Serum Iron, TIBC <0.01	Significant association
Malla et al., 2015 [15]	Nepal	6-60 months	92 /70	Simple	Hb, MCV, MCHC, RDW, Serum Iron-0.001; MCHC- 0.005; TIBC-0.01; Serum Ferritin-0.035	Significant association
Subbarao et al., 2019 [16]	India	6 months-6 years	100/100	Not mentioned	Hb-0.03, MCV-0.12, MCH-0.506, RDW-0.00, Serum Ferritin-0.00	Significant association
Tripathy et al., 2020 [17]	India	6-60 months	240/100	Simple	Hb-0.35, MCV-0.77, MCHC-0.35, RDW-0.5, Serum Iron-0.16, TIBC-0.59, Serum Ferritin-0.28	No association
Awais et al., 2022 [18]	Pakistan	6-59 months	95/95	Not mentioned	IDA-0.326; p-value of Hb, Hct, Serum Ferritin not measured	insignificant association but a slightly positive likelihood of febrile fits with IDA
Jang et al., 2019 [19]	South Korea	6-60 months	63/65.	Both simple and complex	Hb-0.77, Hct-0.72, MCV-0.75, MCH-0.95, MCHC-0.49, RDW-0.49, Serum Iron-0.01, TIBC-0.05, Serum Ferritin <0.001, Transferrin Saturation-0.01	Iron deficiency is associated but IDA is not associated
Yarigarravesh et al., 2021 [20]	Iran	6 months-6 years	60/60	Simple	Hb, HCT, Serum Ferritin, Serum Iron, TIBC >0.05; IDA >0.05	No association
Chaudhary et al., 2021 [21]	Nepal	6-60 months	68/68	Both simple and complex	Hb, MCV-0.01; MCH-0.26; MCHC-0.45; TIBC-0.03; Serum Ferritin-0.04; Transferrin saturation, Serum Iron-0.00, IDA-0.01	Significant association
Shaikh et al., 2018 [22]	India	6-60 months	50/50	Not mentioned	Hb-0.44; MCV-0.06; MCH-0.93; RDW-0.231; TIBC-0.036; Serum Ferritin-0.27; Serum Iron-0.83	No association
Mallela et al., 2022 [23]	India	6-60 months	52 /52	Both simple and complex	Hb, Serum Ferritin-0.001; Serum Iron-0.2	Significant association
Kumari et al., 2023 [24]	India	1-5 years	50/50	Simple	Hb, MCV, MCH, RDW, TIBC, Serum Iron, Serum ferritin <0.001	Significant association
Sarmad et al., 2020 [25]	Pakistan	6-60 months	60/60	Simple	Hb-0.001; RDW, Serum Ferritin-0.002; IDA-0.001	Significant association
Shankar et al., 2019 [26]	India	6-60 months	104/104	Not mentioned	Hb, MCV, Transferrin saturation%, Serum Iron, Serum Ferritin <0.001	Significant association
Elkafafy et al., 2021 [27]	Egypt	6-60 months	50/50	Simple	Hb, MCHC-0.001, MCH-0.003, RDW-0.004, TIBC, Serum Ferritin, IDA <0.001, Serum Iron-0.01	Significant association

Discussion

Type of FS and IDA

A simple FS was defined as a primary generalized tonic-clonic seizure accompanied by a fever, lasting for a maximum of 15 minutes, and not recurring within 24 hours with no preexisting neurological disease. In contrast, a complex FS was characterized by a longer duration (more than 15 minutes), a focal onset, previous neurological abnormality, and the possibility of recurrence within 24 hours [2].

Four studies unanimously showed that simple FS is more common than complex FS [8,19,21,23]. In a study by Chaudhary et al., no association was suggested between the type of FS and IDA with a p-value of 0.77 [21]. Similarly, Jang et al. reported no discernible variations in hematological parameters, including hemoglobin (Hb), serum iron, total iron-binding capacity (TIBC), ferritin, or transferrin saturation, when comparing simple FS and complex FS [19]. The most recent study by Kumari and her team yielded comparable findings of mean hemoglobin. However, this disparity was not statistically significant (p-value <0.27). Similarly, the mean levels of mean corpuscular volume (MCV) and mean corpuscular hemoglobin (MCH) in individuals with simple FS were 65.8 fL and 22.8 pg, respectively, whereas, in those with complex type, these values were 70.9 fL and 23.9 pg, respectively. Again, these differences were not found to be statistically significant [24].

Red Blood Cell Indices and Its Significance With FS

MCV is a laboratory parameter to assess the average size and volume of red blood cells [28]. In cases of chronic iron-deficient anemia, the insufficient iron supply can lead to defects in the protoporphyrin rings within heme molecules, resulting in a reduction in MCV levels (<70 fL), which is characteristic of IDA [16]. MCH blood test measures the quantity of hemoglobin in red blood cells. In cases of IDA, the MCH tends to be reduced, and a value below 23 pg is considered noteworthy [29]. Mean corpuscular hemoglobin concentration (MCHC) measures the average hemoglobin concentration per unit volume of red blood cells [29]. The diversity observed in the size of red blood cells is assessed by a test named red cell distribution width (RDW) [29]. A high RDW means they differ in size more significantly, a characteristic of IDA [29]. Among the 23 studies analyzed, five of which were conducted by Sharif et al., Yarigarravesh et al., Shaikh et al., Jang et al., and Tripathy et al. reported that Hb exhibited no statistically significant association, with corresponding p-values of 0.06, 0.37, 0.44, 0.77, and 0.35, respectively [10,17,19,20,22]. The statistical significance of mean Hb levels was observed in all the studies except the one mentioned earlier [5-9,11-16,21,23-27].

Out of the 18 studies that conducted MCV measurements, 13 of them reported that the mean MCV value was lower in the FS group when compared to the group with febrile illnesses but no seizures with p-value <0.005 [5-7,9,11-15,21,24,26,27]. Across the majority of studies, it was observed that the mean MCH and MCHC values were lower in the FS group when compared to those with febrile illness and no seizures. However, statistical significance was found for MCH in only eight out of the 16 studies that measured it [5,7,9,11,13,15,24,27], and for MCHC, it was significant in five out of the 10 studies that examined it [5,7,9,15,27].

The average RDW value in the FS cluster was higher than what was observed in individuals with fever but no seizures. However, this variant did not achieve statistical significance in three of the 13 studies that measured it [17,19,22].

Iron Indices and Its Significance With FS

Ferritin serves as a highly reliable indicator of one's iron stores. Diminished serum ferritin levels are the key feature of absolute iron deficiency, signifying depleted iron reserves. Levels below 30 mg/L are the commonly accepted threshold for identifying mild cases, and when anemia is present, ferritin levels usually dip even further, often falling below 10-12 mg/L. In the absence of inflammation or infections, serum ferritin demonstrates the strongest association with bone marrow stainable iron levels, previously considered the gold standard for assessing iron store depletion [30].

Indicators that align with the presence of iron deficiency encompass several vital parameters. These include a diminished serum iron level, a reduced transferrin saturation, and an elevated total iron-binding capacity (TIBC). When these values manifest in tandem, they collectively point toward a state of iron deficiency, underscoring the importance of considering multiple facets of iron metabolism in the diagnostic process [31]. Assessing transferrin saturation below 16% is not essential for diagnostic purposes, yet it offers diagnostic significance in cases of functional deficiency where serum ferritin lacks reliability [30].

A predominant trend emerges in the comprehensive analysis of articles within this research, strongly supporting the significant relationship between ferritin levels and FS [5-9,11,12,19,21,24,26,27]. Nevertheless, there were notable exceptions among the findings. In the study conducted by Tripathy et al. involving 340 children, the calculated odds ratio with a confidence interval of 1.31 (0.797-2.159) yielded a statistically insignificant p-value of 0.28 in relation to mean serum ferritin levels [17]. Similarly, in the investigation by Yarigarravesh et al., the p-value associated with mean ferritin levels was statistically insignificant [20]. Furthermore, the study led by Shaikh et al. revealed that ferritin levels exhibited low values in only 2% of children with FS, in contrast to 0% in children with febrile illness but no seizures. This result suggests a lack of statistical significance with a p-value of 0.27 [22].

Among the studies that assessed serum iron levels, all except three, namely, Tripathy et al., Yarigarravesh et al., and Shaikh et al. [17,20,22] demonstrated a statistically significant association between iron levels and IDA [5,7,8,10-12,14,15,19,21,24,26,27]. In a study conducted by Madavi et al., mean serum iron levels were significantly lower in children with FS when compared to those without [7]. This trend was corroborated by several other studies. Furthermore, in research led by Jang et al., low serum iron levels below 22 ng/dL were linked to an increased risk of developing FS, with an odds ratio of 3.42 and a 95% confidence interval ranging from 1.31 to 8.9, accompanied by a p-value of 0.012 [19]. In a study by Shankar et al., serum iron levels below 60 µg/dL were observed in 52.9% of children who experienced FS, as opposed to 14.4% in children with fever and no seizures [26].

The average values of TIBC exhibited statistically significant differences between the febrile convulsion group and the fever without convulsion group in the majority of studies [7-12,14,15,21,22,27]. However, contrary findings were observed in the research conducted by Tripathy et al. and Yarigarravesh et al. [17,20]. The mean transferrin saturation percentage was significantly lower by 7.93% in the FS group compared to the fever without seizures group in a study by Chaudhary et al., a trend consistently reflected in other studies [5,8,10,21,26]. In the study by Sharif et al., insignificant differences between the two groups were noted in the parameter above [10]. Jang et al. reported transferrin saturation percentage to be significantly associated with an increased risk of FS with a p-value of 0.01 [19].

IDA and FS

As iron deficiency advances, a series of biochemical and hematologic events unfold in a sequential manner. Initially, tissue iron stores are depleted, which is indicated by a decrease in serum ferritin. Subsequently, serum iron levels decline, serum TIBC increases, and transferrin saturation falls below the normal range. As iron stores decrease, iron becomes unavailable to combine with protoporphyrin to produce heme. This results in an accumulation of free erythrocyte protoporphyrins and disrupts hemoglobin production. At this stage, iron deficiency progresses to iron deficiency anemia [32].

A study in Nepal shows an odds ratio of IDA for the group of FS to be 2.5 times that of the fever group without convulsion [21]. Similarly, two studies conducted in India showed anemia due to iron deficiency significantly increased the risk of experiencing FS. The odds ratio of Sit et al. and Bhat et al. was 6.3 and 5.1, respectively, with a 95% confidence interval [9,13]. A study by Madavi et al. in 2021 demonstrated an odds ratio of 8.4, the highest among all the studies included [7]. Saleem et al. conducted a study where 88% of FS had IDA compared to those who had only febrile illness, where it was 24% [14]. Furthermore, various other studies showed similar results [5,6,8,10-12,15,16,20,23,25-27]. Contrasting results were obtained in a study by Shaikh et al. where no statistical significance was seen between IDA and FS with a p-value of 0.44 [22]. Research in South Korea indicated that having insufficient iron, defined as having a low ferritin level (<30 ng/mL) or low serum iron (<22 ng/dL), was linked to a higher likelihood of experiencing FS. Interestingly, the presence of IDA didn't show a significant connection to FS. These discrepancies in the relationship between FS and anemia or iron deficiency may be due to variations in ethnic backgrounds, socioeconomic status, nutritional conditions, and the differing criteria used to define anemia and iron deficiency in various studies [19]. A study by Awais et al. in Pakistan concluded an insignificant association but a positive likelihood of IDA with febrile fits with an odds ratio of 1.475 and a p-value of 0.326 [18]. Similar results were obtained in a study in January 2020 in India, where the frequency of IDA with FS was more than the group without fits but was not statistically significant enough to establish as a risk factor for FS [17].

Limitations

There was a variation in the study data between observational studies ranging from the sample size, ethnicity of participants, prevalence of anemia, definition of IDA, socio-economic status, and nutritional state. Moreover, this article lacked clinical trial articles in a language other than English. Also, studies published before 2013 were excluded, which might have impacted the result of an association between IDA and FS. Furthermore, some research studies did not encompass the complete array of iron indices, which could have provided additional insights into the connection between iron indices and FS. Additionally, the quality of one of study is insufficient.

## Conclusions

In summary, our comprehensive review of the available evidence indicates a notable link between IDA and an increased risk of FS in children. Iron deficiency can be regarded as a potential risk factor for FS. While the prevalence of IDA was more common in simple FS than in complex ones, the association of IDA with either type of FS does not appear statistically significant. To ascertain whether iron supplementation can effectively prevent the occurrence of FS, further prospective studies involving larger population samples are necessary.
